# Supplementary material for: The contribution of X-linked coding variation to severe developmental disorders
Source: Nat Commun. 2021 Jan 27;12:627. doi: 10.1038/s41467-020-20852-3 (PMC7840967; doi:10.1038/s41467-020-20852-3)
Supplement: Supplementary file 1 — Supplementary Information [file 41467_2020_20852_MOESM1_ESM.pdf]

Supplementary Figures

Supplementary Figure 1: Histogram of the number of affected organ systems in male versus female probands in DDD. We followed the procedure in [1] when counting affected organ systems, to avoid double-counting HPO terms that fall under multiple organ systems.

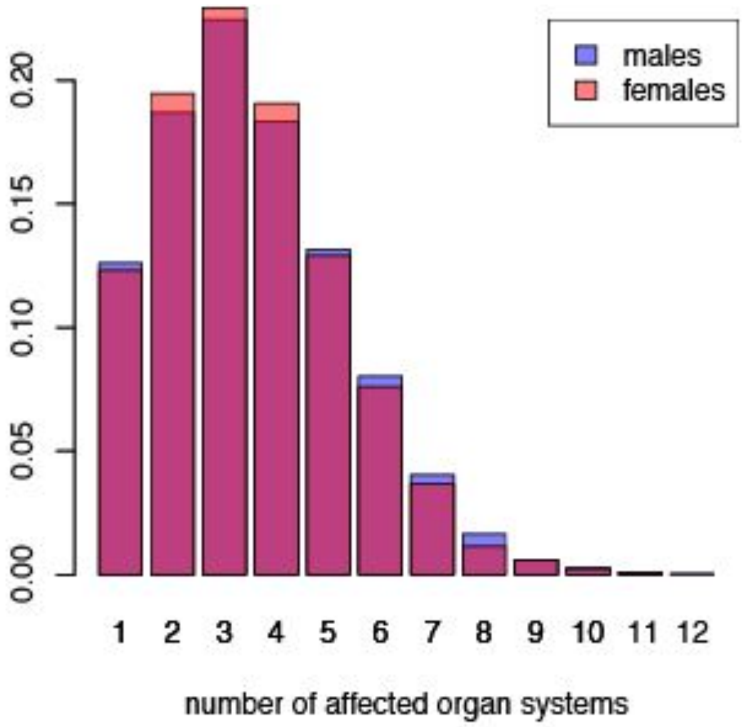

Supplementary Figure 2: Histograms indicating the distribution of bootstrapped values for metrics reported in the main text, from 10,000 iterations. See section on "Burden analysis" in the Methods for details. The red lines indicate the observed values, purple lines the 95% confidence interval from bootstrapping, and orange line in panel (D) is the value expected under Haldane's theory. (A) The proportion of the excess (observed-expected for PTV and missense/inframe variants) in males that is in known genes. (B) The proportion of the excess in females that is in known genes. (C) The proportion of the excess in males that is *de novo*, across all genes. (D) The proportion of the excess in males that is *de novo*, in X-linked recessive genes. Note that (D) includes only PTV and missense SNVs, since the male:female mutation rate ratio is not known for indels. The x-axis has been truncated in panels C and D for easier visualisation.

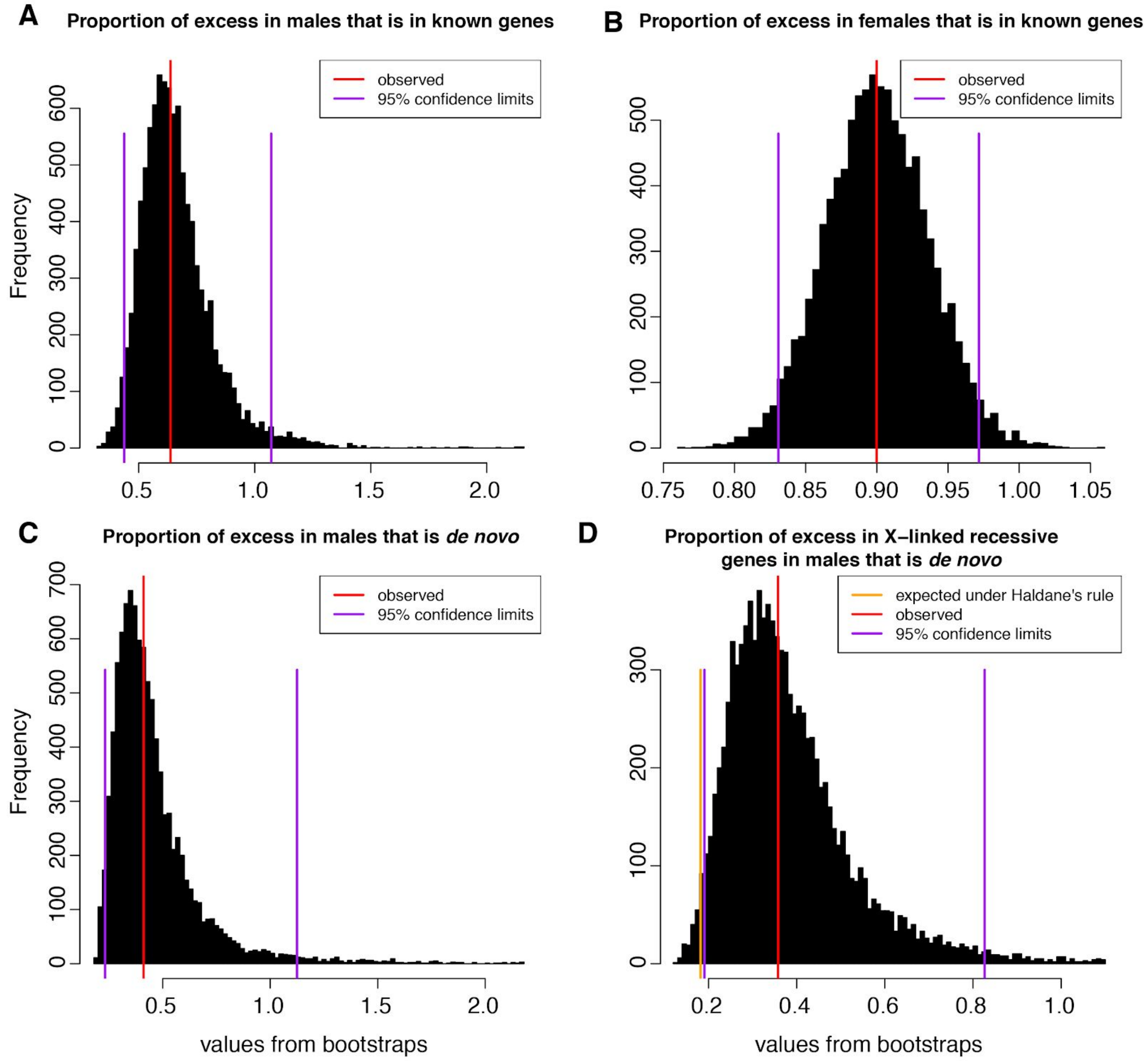

Supplementary Figure 3: Positive predictive values estimated for inherited variants in trio probands with different filters. Error bars show 95% confidence intervals calculated as described in the Methods.

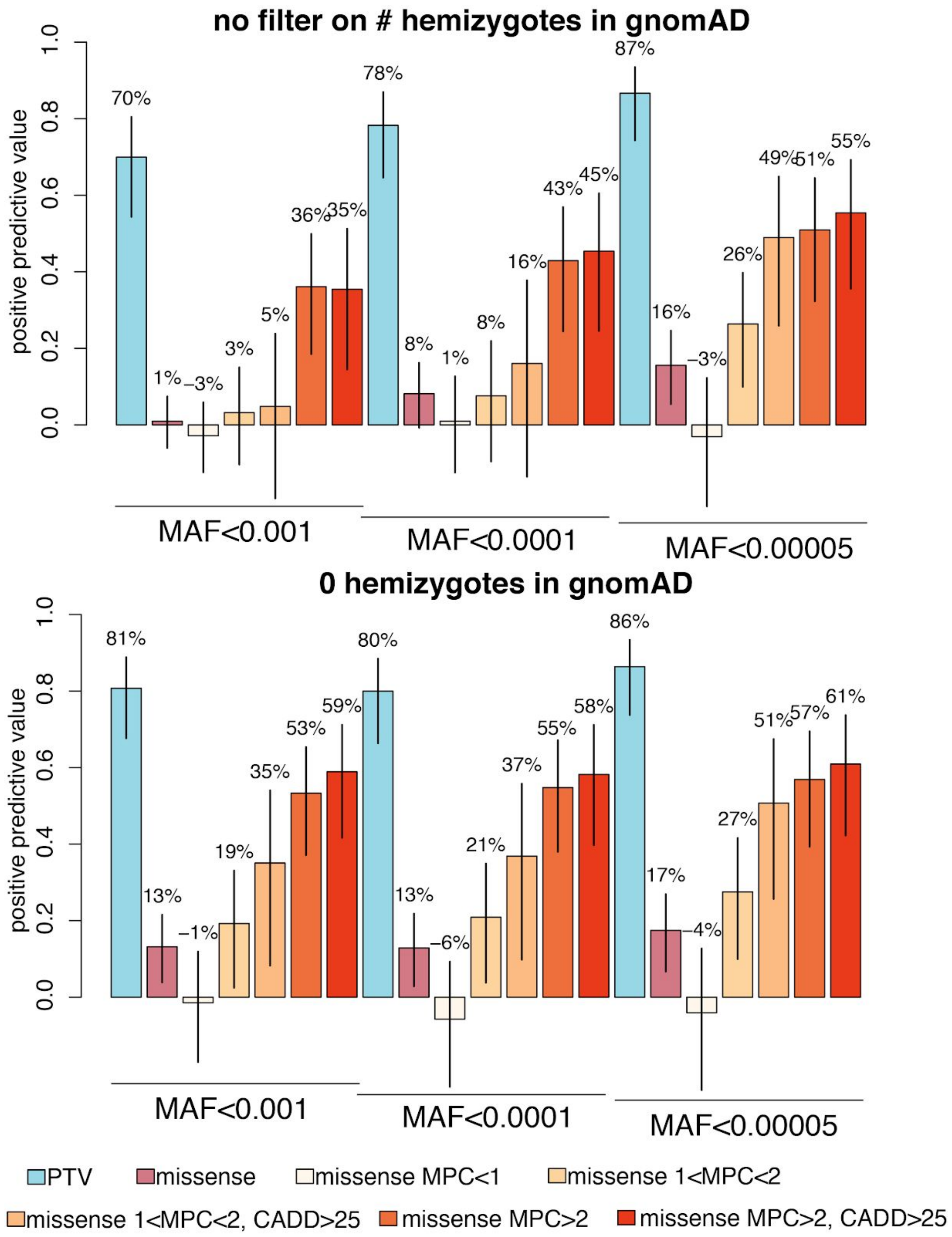

Supplementary Figure 4: Assessing cognition and fertility in female carriers of rare variants in XLR DD-associated genes in UK Biobank. (A) Total number of rare variants in XLR genes passing the filters described in the Methods. (B-C) Point estimates of effect size of these variants on fluid intelligence (B) and number of live births (C), with 95% confidence intervals. These effects were estimated in a linear regression correcting for age and the top 10 genetic principal components.

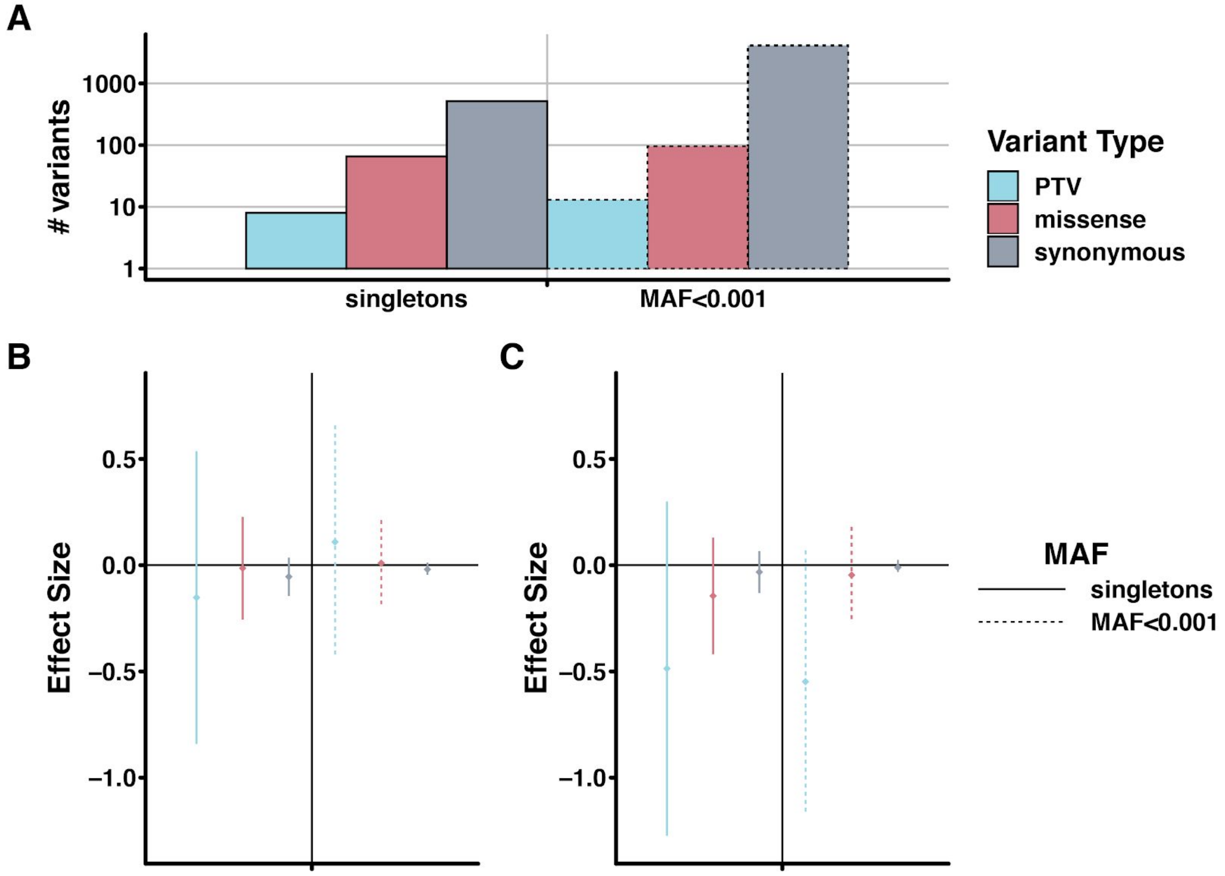

Supplementary Figure 5: Positive predictive value for different classes of variants in X-linked recessive versus X-linked dominant genes. This excludes genes that were classed as both X-linked dominant and X-linked recessive. Error bars show 95% confidence intervals calculated as described in the Methods.

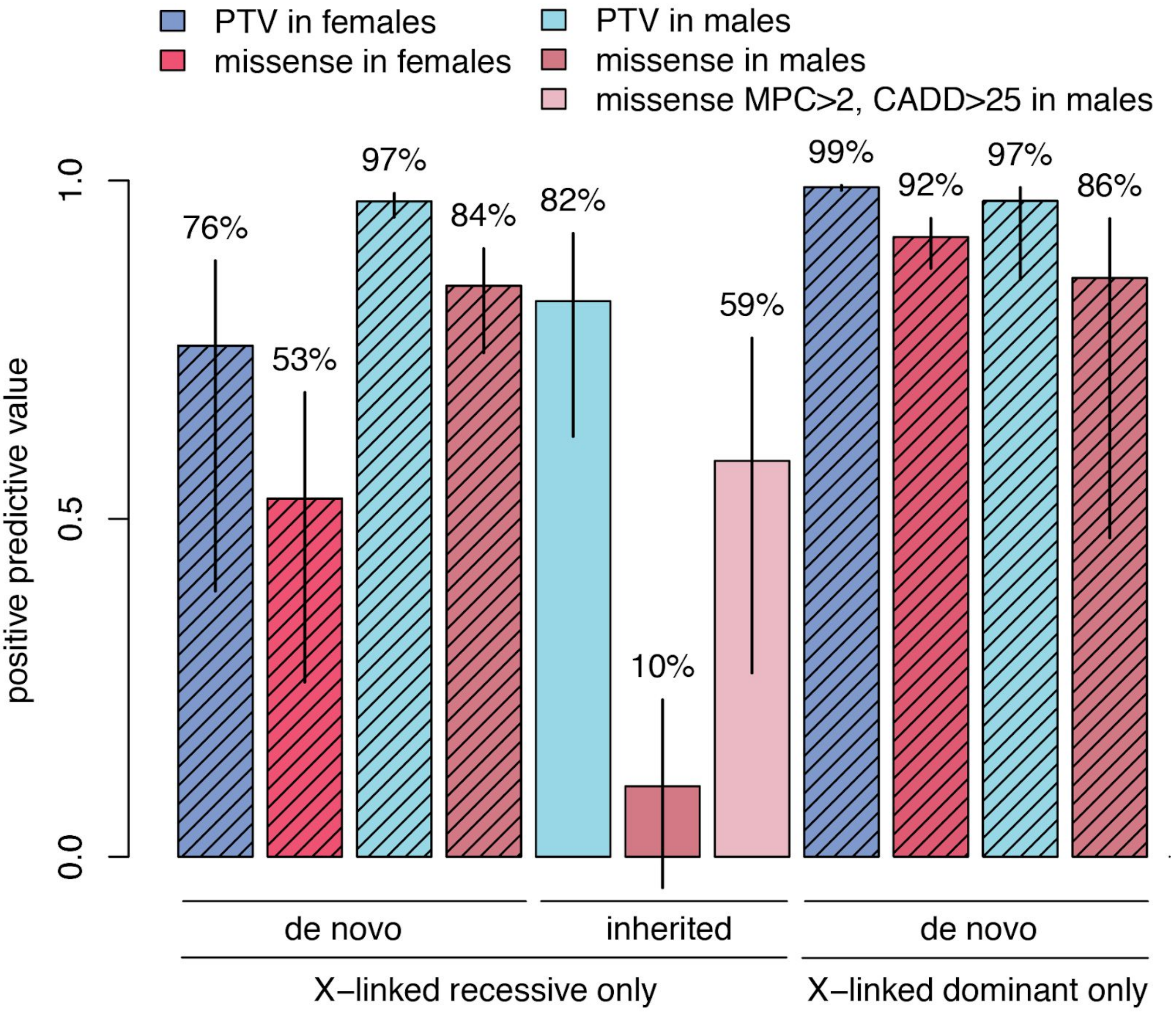

Supplementary Tables

Supplementary Table 1: Results from comparing growth metrics and developmental milestones between males and females in DDD using linear regression. The estimate shown is the effect size for being male, and S.E. is the standard error. The p-value is a one-sided p-value from the z-test in the linear regression (no adjustment for multiple testing). Probands were excluded if they had missing data or, for the milestones, had not yet reached the milestone at the time of assessment. OFC: occipital frontal circumference. SD: standard deviation of the population distribution for that sex.

**Milestone or measurement**	**# probands included**	**unconditional**			**after conditioning on age at assessment**		
		**Estimate**	**S.E.**	**p-value**	**Estimate**	**S.E.**	**p-value**
age at assessment (years)	13,443	-0.402	0.108	2.08E-04	-	-	-
age at talking (months)	6,660	0.248	0.354	4.83E-01	0.428	0.349	2.21E-01
age at walking (months)	9,362	-1.297	0.231	2.14E-08	-1.195	0.231	2.41E-07
birthweight (SD)	12,373	0.019	0.026	4.62E-01	0.019	0.026	4.60E-01
OFC at birth (SD)	2,037	-0.037	0.100	7.09E-01	-0.040	0.100	6.89E-01
height (SD)	10,256	0.273	0.050	5.77E-08	0.270	0.050	7.50E-08
weight (SD)	10,471	0.649	0.613	2.89E-01	0.633	0.613	3.01E-01
OFC at recruitment (SD)	11,515	0.378	0.047	1.27E-15	0.395	0.047	4.49E-17

Supplementary Table 2: Rare PTVs in consensus hemizygous genes among WES females recruited to UK Biobank. Position coordinates are given for build GRCh38 of the human reference genome and all fall on the X chromosome. The allele count in UK Biobank is out of a total of 53,445 X chromosomes . All variants were absent from gnomADv3 [2] and ClinVar [3].

position	reference allele	alternate allele	Gene	ENSEMBL Gene ID	CADD	Allele Count
68064091	G	A	*OPHN1*	ENSG00000079482	36	1
7257241	G	A	*STS*	ENSG00000101846	26.2	2
7257241	G	A	*STS*	ENSG00000101846	26.2	2
7257357	C	T	*STS*	ENSG00000101846	35	2
24742069	AC	A	*POLA1*	ENSG00000101868	26.9	1
24727781	G	A	*POLA1*	ENSG00000101868	31	1
110688651	A	AC	*CHRDL1*	ENSG00000101938	31	1
110681651	T	C	*CHRDL1*	ENSG00000101938	27.6	2
110688729	C	A	*CHRDL1*	ENSG00000101938	37	1
110681651	T	C	*CHRDL1*	ENSG00000101938	27.6	2
131275692	C	T	*IGSF1*	ENSG00000147255	37	1
80681410	G	A	*BRWD3*	ENSG00000165288	37	1
71124843	CAGTA	C	*MED12*	ENSG00000184634	27.2	1

Supplementary Table 3: Results of tests for a depletion of DNMs in males compared to females, likely due to male lethality. Only genes that were genome-wide significant are included. The p-value is from a one-sided binomial test comparing the observed fraction of DNMs that are observed in males to the fraction expected given the relative mutation rates corrected for coverage (see Methods).

gene	DNMs in males		DNMs in females		fraction observed in males	binomial p-value
	PTV	missense/ inframe	PTV	missense/ inframe		
DDX3X	0	2	21	27	0.04	0.002
MECP2	0	1	11	13	0.04	0.025
HDAC8	0	0	5	8	0.00	0.055
SMC1A	0	0	9	4	0.00	0.055
NAA10	0	0	0	8	0.00	0.138
CDKL5	0	0	3	4	0.00	0.168
STAG2	0	0	3	3	0.00	0.206
PDHA1	0	0	3	2	0.00	0.255
USP9X	0	1	7	2	0.10	0.290
HNRNPH2	0	1	1	7	0.11	0.343
MED12	0	1	1	7	0.11	0.343
ZC4H2	0	1	5	1	0.14	0.479
KIAA2022	1	0	4	0	0.20	0.500
MSL3	1	0	3	0	0.25	0.500
WDR45	1	3	12	2	0.22	0.500
KDM6A	2	1	2	3	0.38	0.729
CNKSR2	2	0	2	0	0.50	0.768
CASK	1	4	6	3	0.36	0.813
HUWE1	0	6	0	9	0.40	0.910
PHF8	2	1	0	1	0.75	0.973
IQSEC2	4	4	5	4	0.47	0.985
UPF3B	3	0	0	0	1.00	0.994
KDM5C	3	2	1	1	0.71	0.996

Supplementary Table 4: Results from logistic regressions testing the difference in polygenic scores for the indicated traits between 216 males who were suspected by clinicians to have X-linked inheritance versus 3439 who were not. We excluded males with a potentially diagnostic X-linked variant.

trait	effect size	standard error	p-value
neurodevelopmental disorders [4]	-0.003	0.066	0.969
IQ [5]	-0.057	0.071	0.426
schizophrenia [6]	-0.009	0.070	0.903
educational attainment [7]	-0.026	0.070	0.712

Supplementary Table 5: Parameters used for TADA. RR is the relative risk of causal mutations; β is a parameter in its prior distribution. DNM: *de novo* mutation model, c/c: case/control inherited model.

	PTV				missense/inframe			
π	RR_{DNM}	β_{DNM}	$RR_{c/c}$	$\beta_{c/c}$	RR_{DNM}	β_{DNM}	$RR_{c/c}$	$\beta_{c/c}$
0.01	860.762	1	73.062	1	222.877	1	6.150	1.000
0.05	172.952	1	15.412	1	45.375	1	2.030	4.811
0.1	86.976	1	8.206	1	23.188	1	1.515	6.496
0.15	58.317	1	5.804	1	15.792	1	1.343	7.355
0.2	43.988	1	4.603	1	12.094	1	1.257	7.876
0.25	35.390	1	3.882	0.283	9.875	1	1.206	8.225

Supplementary References

1. Martin, H. C. *et al.* Quantifying the contribution of recessive coding variation to developmental disorders. *Science* **362**, 1161–1164 (2018).
2. Karczewski, K. J. *et al.* The mutational constraint spectrum quantified from variation in 141,456 humans. *Nature* **581**, 434–443 (2020).
3. Landrum, M. J. *et al.* ClinVar: improving access to variant interpretations and supporting evidence. *Nucleic Acids Res.* **46**, D1062–D1067 (2018).
4. Niemi, M. E. K. *et al.* Common genetic variants contribute to risk of rare severe neurodevelopmental disorders. *Nature* **562**, 268–271 (2018).
5. Sniekers, S. *et al.* Genome-wide association meta-analysis of 78,308 individuals identifies new loci and genes influencing human intelligence. *Nat. Genet.* **49**, 1107–1112 (2017).
6. Pardiñas, A. F. *et al.* Common schizophrenia alleles are enriched in mutation-intolerant genes and in regions under strong background selection. *Nat. Genet.* **50**, 381–389 (2018).
7. Lee, J. J. *et al.* Gene discovery and polygenic prediction from a 1.1-million-person GWAS of educational attainment. *Nat. Genet.* **50**, 1112 (2018).
