# Supplementary material for: The contribution of X-linked coding variation to severe developmental disorders
Source: Nat Commun. 2021 Jan 27;12:627. doi: 10.1038/s41467-020-20852-3 (PMC7840967; doi:10.1038/s41467-020-20852-3)
Supplement: Supplementary file 3 — Description of Additional Supplementary Files [file 41467_2020_20852_MOESM3_ESM.pdf]

Description of Additional Supplementary Files

File Name: Supplementary Data 1.

Description: Results from logistic regression comparing the prevalence of different phenotypic features amongst males versus females in DDD, controlling for age at assessment.

File Name: Supplementary Data 2.

Description: De novo mutations that passed our filtering and were used in the burden analysis and gene-based tests. Filtering is described in the Methods section ("Exome sequencing, variant annotation and variant quality control"). pp_dnm: posterior probability of being a DNM from DeNovoGear.

File Name: Supplementary Data 3.

Description: Variant counts and results from gene-based tests using TADA to analyse de novo and inherited variants in males, or de novo enrichment tests in males and females. We have indicated the inheritance mode(s) from DDG2P for all genes that are 'confirmed' or 'probable' DDG2P genes, and separately for all 'possible' DDG2P genes. We have also indicated the inheritance mode/s annotated in OMIM for genes that are also in DDG2P. The genes highlighted in yellow passed our genome-wide significance threshold.
