# Supplementary material for: The contribution of X-linked coding variation to severe developmental disorders
Source: Nat Commun. 2021 Jan 27;12:627. doi: 10.1038/s41467-020-20852-3 (PMC7840967; doi:10.1038/s41467-020-20852-3)
Supplement: Supplementary file 7 — Reporting summary [file 41467_2020_20852_MOESM7_ESM.pdf]

nature research

Corresponding author(s): Hilary Martin

Last updated by author(s): Apr 8, 2020

Reporting Summary

Nature Research wishes to improve the reproducibility of the work that we publish. This form provides structure for consistency and transparency in reporting. For further information on Nature Research policies, see Authors & Referees and the Editorial Policy Checklist .

Statistics

For all statistical analyses, confirm that the following items are present in the figure legend, table legend, main text, or Methods section.

n/a	Confirmed	
☐	☒	The exact sample size (*n*) for each experimental group/condition, given as a discrete number and unit of measurement
☒	☐	A statement on whether measurements were taken from distinct samples or whether the same sample was measured repeatedly
☐	☒	The statistical test(s) used AND whether they are one- or two-sided<br>*Only common tests should be described solely by name; describe more complex techniques in the Methods section.*
☐	☒	A description of all covariates tested
☐	☒	A description of any assumptions or corrections, such as tests of normality and adjustment for multiple comparisons
☐	☒	A full description of the statistical parameters including central tendency (e.g. means) or other basic estimates (e.g. regression coefficient) AND variation (e.g. standard deviation) or associated estimates of uncertainty (e.g. confidence intervals)
☐	☒	For null hypothesis testing, the test statistic (e.g. *F*, *t*, *r*) with confidence intervals, effect sizes, degrees of freedom and *P* value noted<br>*Give P values as exact values whenever suitable.*
☒	☐	For Bayesian analysis, information on the choice of priors and Markov chain Monte Carlo settings
☒	☐	For hierarchical and complex designs, identification of the appropriate level for tests and full reporting of outcomes
☒	☐	Estimates of effect sizes (e.g. Cohen's *d*, Pearson's *r*), indicating how they were calculated

Our web collection on statistics for biologists contains articles on many of the points above.

Software and code

Policy information about availability of computer code

Data collection	No software was used to collect data.
Data analysis	Mapping of short-read sequences for each sequencing lanelet was carried out using the Burrows-Wheeler Aligner versions 0.5.10 using both backtrack (aln) algorithm and the versions 0.7.12 using the bwa-mem algorithm, with the GRCh37 1000 Genomes Project phase 2 reference (also known as hs37d5). Sample-level BAM improvement was carried out using the Picard Markduplicates (versions 1.98 and 1.114) and Genome Analysis Toolkit IndelRealigner (GATK version 3.1.1 and version 3.5.0), which performs realignment of reads around known and discovered indels (insertions and deletions). Single-nucleotide variants (SNVs) and indels were called using the GATK HaplotypeCaller, CombineGVCFs and GenotypeGVCFs (GATK version 3.5.0). Bcftools (version 1.8-30-gb717d08) and custom Perl (version 5) scripts were used to filter the variants for the case/control analysis. De novo mutations were called with DeNovoGear (version 0.10.18) and filtered using custom Python (version 3.5.0) scripts. Variant Effect Predictor (with Ensembl build 38) was used for variant annotation. All statistical analyses were carried out in R (version 3.4.0), and the scripts have been released here https://github.com/hilarymartin/DDD_chrX.

For manuscripts utilizing custom algorithms or software that are central to the research but not yet described in published literature, software must be made available to editors/reviewers. We strongly encourage code deposition in a community repository (e.g. GitHub). See the Nature Research guidelines for submitting code & software for further information.

Data

Policy information about availability of data

All manuscripts must include a data availability statement. This statement should provide the following information, where applicable:

- Accession codes, unique identifiers, or web links for publicly available datasets
- A list of figures that have associated raw data
- A description of any restrictions on data availability

The full variant call files used in this study are accessible in the European Genome-Phenome Archive as dataset EGAD00001004389, and a file of phenotypic and family descriptions under EGAD00001004388. Both of these are under managed access to ensure that the work proposed by the researchers is allowed under the study's ethical approval. The de novo mutations used in the analysis are in Supplementary Data 2. Databases used in this study: Online Mendelian Inheritance in Man https://omim.org/; ClinVar https://www.ncbi.nlm.nih.gov/clinvar/; Developmental Disorder Gene-to-Phenotype list https://www.ebi.ac.uk/gene2phenotype/downloads; GRCh37 1000 Genomes Project phase 2 reference (hs37d5) https://www.ncbi.nlm.nih.gov/assembly/GCF_000001405.13/.

Field-specific reporting

Please select the one below that is the best fit for your research. If you are not sure, read the appropriate sections before making your selection.

- [x] Life sciences
- [ ] Behavioural & social sciences
- [ ] Ecological, evolutionary & environmental sciences

For a reference copy of the document with all sections, see nature.com/documents/nr-reporting-summary-flat.pdf

Life sciences study design

All studies must disclose on these points even when the disclosure is negative.

Sample size	No sample size calculation was used. The sample size was limited by the number of patients we managed to recruit to the DDD study. We state clearly in the Discussion that with this sample size, some of the gene-based tests were underpowered, and confidence intervals for many estimates are still large. However, to our knowledge, this is still the largest study of its kind available to date.
Data exclusions	Sample exclusions are described in the Methods under "Sample QC". Variant quality control was performed separately for the case/control analysis and the de novo mutation analysis, and is described in the Methods section under "Exome sequencing, variant annotation and variant quality control".
Replication	No formal replication was attempted since similar datasets of an appropriate size are scarce and difficult to gain access to. However, we note in the Discussion how our findings compare to previous smaller studies.
Randomization	Randomisation was not relevant to the study since no experiments were involved.
Blinding	Blinding was not relevant to the study since no experiments were involved.

Reporting for specific materials, systems and methods

We require information from authors about some types of materials, experimental systems and methods used in many studies. Here, indicate whether each material, system or method listed is relevant to your study. If you are not sure if a list item applies to your research, read the appropriate section before selecting a response.

Materials & experimental systems

n/a	Involved in the study	
x		Antibodies
x		Eukaryotic cell lines
x		Palaeontology
x		Animals and other organisms
	x	Human research participants
x		Clinical data

Methods

n/a	Involved in the study	
x		ChIP-seq
x		Flow cytometry
x		MRI-based neuroimaging

Human research participants

Policy information about studies involving human research participants

Population characteristics	There were 7,843 male and 5,619 female probands in the DDD study, and all of these were used for the phenotypic comparisons shown in Supplementary Table 1 and Supplementary Figure 1. The ages of the probands at recruitment were: mean 7.14 years for males (standard deviation 5.96); mean 7.54 years for females (standard deviation 6.48 years). The final analysis of genetic data was conducted on 7,138 male probands (5,138 in trios), 3,908 female probands in trios, and 8,551 unaffected fathers.
Recruitment	Individuals with severe, undiagnosed developmental disorders were recruited, along with their parents, to the DDD project by 24 clinical genetics centres within the United Kingdom National Health Service and the Republic of Ireland. They had to have at least one of the following characteristics:<br>1. Neurodevelopmental disorder – for example developmental delay and/or learning disability (of a level requiring or likely to require a statement of special educational needs), epileptic encephalopathy or cerebral palsy<br>2. Congenital anomalies – multiple congenital anomalies (two or more major anomalies) or a single major anomaly together with a neurodevelopmental disorder, aberrant growth, dysmorphic features or unusual behaviour<br>3. Abnormal growth parameters (height, weight, head circumference (OFC)) – two or more parameters >3SD above or below the mean or a single parameter >4SD above or below the mean (except for obesity where the threshold for isolated obesity is >4.5SD together with a strong suspicion of a genetic aetiology)<br>4. Unusual behavioural phenotype in conjunction with one or more of the above features or extreme behavioural phenotype strongly suspected to have a genetic basis (including classical autism)<br>5. Genetic disorder of significant impact for which the molecular basis is currently unknown with: (i) several affected family members or (ii) one other affected family member with a rare, consistent and distinctive phenotype or (iii) a single case that is associated with a severe phenotype.<br><br>By definition, these patients could not have had a genetic diagnosis already through the usual clinical means. We discuss in the manuscript how this might mean that patients with obvious X-linked inherited causes were depleted in our cohort, and we emphasise in the Discussion that the estimates we present for attributable fraction, fraction of de novo causes etc. are specific to this cohort and may not necessarily extend to other cohorts with e.g. different recruitment criteria.
Ethics oversight	The study has UK Research Ethics Committee approval (10/H0305/83, granted by the Cambridge South Research Ethics Committee and GEN/284/12, granted by the Republic of Ireland Research Ethics Committee).

Note that full information on the approval of the study protocol must also be provided in the manuscript.
